# Knobbed acrosome defect is associated with a region containing the genes *STK17b *and *HECW2 *on porcine chromosome 15

**DOI:** 10.1186/1471-2164-11-699

**Published:** 2010-12-09

**Authors:** Anu Sironen, Pekka Uimari, Szabolcs Nagy, Sándor Paku, Magnus Andersson, Johanna Vilkki

**Affiliations:** 1 Agrifood Research Finland, MTT, Biotechnology and Food Research, Genomics, FI-36100 Jokioinen, Finland; 2 University of Pannonia, Institute of Environmental Sciences, H-8200 Veszprem, Hungary; 3 First Department of Pathology and Experimental Cancer Research, Semmelweis University, H-1085 Budapest, Hungary; 4 University of Helsinki, Department of Clinical Veterinary Sciences, Helsinki, Finland

## Abstract

**Background:**

Male infertility is an increasing problem in all domestic species including man. Localization and identification of genes involved in defects causing male infertility provide valuable information of specific events in sperm development. Correct condensation of the sperm head and development of the acrosome are required for fertile sperm. In the Finnish Yorkshire pig population a knobbed acrosome defect (KAD) has been reported which appears to be of genetic origin. In previous studies we have shown that a large number of affected spermatozoa have a cystic swelling anterior to the apical part of the acrosome.

**Results:**

Characterization of the knobbed acrosome affected sperm revealed that both the acrosomal granules and chromatin are affected. This type of KAD appears to be a previously unknown and serious form of the defect. A genome wide scan with PorcineSNP60 Genotyping BeadChip defined the KAD associated region within 0.7 Mbp on porcine chromosome 15. Two genes, *STK17b *and *HECW2*, located within this region were sequenced. The expression of these genes appeared comparable in KA-affected and control boars. The known function of HECW2 in acrosome development highlighted this gene as a good candidate responsible for the KAD. One nonsynonymous SNP was identified within the *HECW2 *gene. However, as this mutation was found in homozygous state in individuals with normal sperm, this is not likely to be the causal mutation.

**Conclusions:**

In this study we identified two candidate genes for a severe defect affecting both the sperm acrosome and chromatin that causes infertility. One of these genes, *HECW2*, plays an important role in ubiquitination, a prerequisite for chromatin remodelling and acrosome formation, highlighting the involvement of this gene in the knobbed acrosome defect and male infertility.

## Background

Male infertility is becoming increasingly prevalent partly due to environmental factors, but many defects in sperm development arise from a genetic cause. Problems in the production and maturation of sperm are the most common causes of male infertility resulting in low sperm numbers, morphologically abnormal sperm or low sperm motility [1-3]. Despite efforts to reveal the genes and their functions in spermatogenesis, little is known about the underlying causes of male infertility. Therefore, the localization and identification of mutations specifically affecting spermatogenesis provide invaluable information for investigating the causes of male infertility.

Mammalian spermatogenesis is a complex process, where diploid spermatogonia develop into haploid, highly specialized spermatozoa. Spermatogenesis includes many testis-specific processes that are controlled by complex regulatory mechanisms [4,5]. During spermiogenesis, haploid round spermatids undergo dramatic biochemical and morphological changes that are governed by specialized gene expression and interactions between various genes and their protein products [6]. Identification of genes involved in sperm development is a prerequisite to understanding the molecular mechanisms of spermatogenesis.

Sperm development is known to be disrupted during spermiogenesis in several acrosomal defects; e.g. globozoospermia in humans, where spermatozoa lack an acrosome [7-9] and the knobbed acrosome defect (KAD) in bulls, boars, stallions, rams, and dogs [10-15]. The acrosome is an organelle that develops over the anterior half of the head in the spermatozoa. It is a cap-like structure derived from the Golgi apparatus. The acrosome contains digestive enzymes, which break down the zona pellucida of the ovum, allowing the sperm to deliver its haploid nucleus into the ova. Disturbances of acrosomal development and function significantly impair the fertilizing capacity of spermatozoa [16].

Knobbed acrosome defect has been recently described in the Finnish Yorkshire pig population [15]. Testicular weights of boars with KAD did not differ from control boars. However, affected boars had a smaller seminiferous tubule diameter and lower number of Sertoli cells relative to control boars [15]. Investigation of the pedigrees of KA-affected boars suggested an autosomal recessive inheritance of the defect. Generally two common boars were identified in the pedigree of the boars with the KAD. Fertility of KA-affected boars is severely compromised. Depending on the amount of knobbed spermatozoa (25-81%) affected boars had poor non-return rate from no pregnancies to 47%, thus KA-affected boars produced no offspring or on average 2.5 fewer piglets per litter than control boars. Here we have characterized further the severity of the sperm head abnormalities in KA-affected boars.

A whole genome scan with microsatellite markers showed increased homozygosity in KA-affected boars in chromosomes 3, 8, 14 and 15 [15]. However, no statistically significant association was detected with available microsatellite markers. In this study we have used the PorcineSNP60 Genotyping BeadChip (Illumina) in order to increase marker density and accurately map the KAD associated region in pigs. All affected boars were homozygous for SNPs covering 432 kb on porcine chromosome 15. The coding region of two genes was located within this homozygous region and sequenced from both a KA-affected and control boar.

## Results

### Microscopical analysis of the KAD

**Figure 1:**
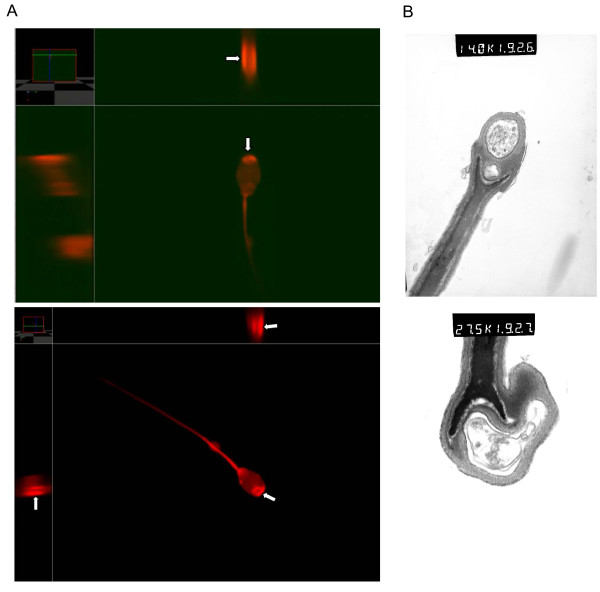
**Microscopical examination of the KA-defect**. A. Confocal laser scanning microscopy images of spermatozoa with acrosomal granule from two KA-affected boars. Yellow lines indicate the perpendicular planes of the 3d-reconstructions. Arrows indicate a vacuolum inside the granule. B. TEM analysis of an acrosomal granule. Two vacuoles are present and the apical part of the nucleus shows a Y-shape.

In the confocal laser scanning microscopy three-dimensional reconstructions of the spermatozoa with acrosomal granules indicated that the granules protruded on both sides and contained a vacuolum (Figure 1A). TEM analyses confirmed the three-dimensional protrusion of the granules and the occurrence of vacuoles within the granules. The nucleus was also shown to be affected as evident from the Y-shaped form at the apical end (Figure 1B) suggesting that the defect affects both the chromatin and acrosome. These findings highlight that this particular and previously unknown KA-defect appears to be a serious form of the acrosomal granule defect.

### SNP quality measures

Based on all available SNPs and the method to estimate IBDs in the Plink software package (pi_hat) the average relatedness among cases and controls was 0.24 and 0.26, respectively. These levels of relatedness are typical in the studied Finnish Yorkshire pig population. The average sample call rate was 95%. There were 2815 SNPs that did not work for any of the samples analysed. Excluding these SNPs, the average SNP call rate was 0.9982 (s.d. = 0.007) and the average minor allele frequency was 0.25 (s.d. = 0.14). Overall, the dataset contained 9216 monomorphic SNPs. Observed distribution of P-values in the Hardy-Weinberg equilibrium test statistics did not differ from expectations. In total, 183 SNPs (excluding SNPs on the X-chromosome) had a P-value <1.0E-06 being lower than expected.

### Genome wide association analysis

**Figure 2:**
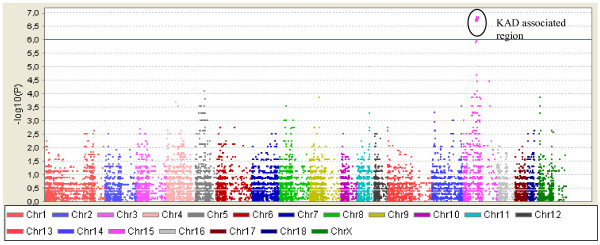
**Manhattan plot of the log10 based P-values across all chromosomes for the KAD in Finnish Yorkshire pig population**. The KA-affected haplotypes of marked KAD associated region supported the expected recessive mode of inheritance and had the lowest P-values.

**Figure 3:**
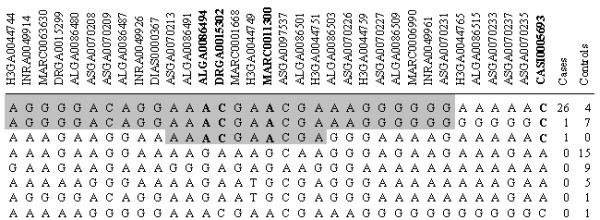
**Haplotype counts in KAD cases and controls on chromosome 15 (91861321-95156189 bp, Pig genome build 9)**. SNPs that are shared among KAD cases are marked in grey background and the SNPs with the smallest P-value from the recessive model are in bold face.

The association test was performed for 47055 SNPs. The Manhattan plot of the log10 based P-values is presented in Figure 2. The recessive model identified a KAD associated region covering approximately 3 Mbp between 93 and 96 Mbp (pig genome build 9) on chromosome 15. After permutation, five SNPs were statistically significant (P-value = 0.0002, Table 1). Four of these SNPs (ALGA0086494, DRGA0015302, MARC0011300, and CASI0005693) are located within a 1.4 Mbp region and were in complete linkage disequilibrium (D' = 1.0, r^2 ^= 1.0). 12 out of 14 KAD cases had inherited two identical copies of the haplotype covering these and other SNPs between them, indicating an extended homozygosity in this region, and thus a common ancestral origin (Figure 3). All KA-affected boars shared a 0.7 Mbp homozygous region between SNPs DIAS0000367 and ALGA0086503 (Figure 3, additional file 1). The CASI0005693 SNP was in stronger linkage disequilibrium with ALGA0086494 and other significant SNPs compared with neighbouring SNPs, highlighting that the position of CASI0005693 may change following a more refined genome build in this region (see additional file 1). The fifth significant SNP (MARC0020403) was located 4 Mbp from the other four SNPs, and was shown to be in linkage disequilibrium (D' = 1.0, r^2 ^= 0.13).

**Table 1:** Genotype counts of cases and controls, nominal, and permutated P-values from the recessive model for the best KAD associated SNPs.

SNP	Chr	Position, bp	Genotypes cases	Genotypes controls	Recessive, P-value	Permutated P-value
ALGA0086494	15	93805621	14/0/0	2/8/11	1.41E-07	0.0002
DRGA0015302	15	93835894	14/0/0	2/8/11	1.41E-07	0.0002
MARC0011300	15	94070930	14/0/0	2/8/11	1.41E-07	0.0002
CASI0005693	15	95156189	14/0/0	2/8/11	1.41E-07	0.0002
MARC0020403	15	90155667	12/2/0	0/7/14	1.66E-07	0.0002

### Candidate genes HECW2 and STK17b

The most promising candidate gene *Ubiquitin-protein ligase E3 *(*HECW2*) was located within the haplotype of two SNPs with the highest P-values; ALGA0086494 and DRGA001532 (Table 1, additional file 1). All KA-affected animals were homozygous for these SNPs and only two control animals had the same homozygote alleles as KA-affected boars (Figure 3). Furthermore, one of these two animals appeared to have a SME-defect and another was removed from breeding at young age due to weak leg conformation and therefore no fresh sperm samples were available for analysis. The SME-defect is a cyst malformation in the sperm head, with indications that this is of acrosomal origin [17,18].

*HECW2 *is expressed in the testis [19] and functions in ubiquitin mediated proteolysis [20]. Ubiquitin signals have been detected during acrosome development [21] and deubiquitinating enzyme mUBPy is upregulated in the testis of wobbler mouse, which is infertile due to the lack of a functional acrosome [22].

Another gene within the KAD homozygous region was *serine/threonine kinase 17b *(*STK17b, DRAK2*, additional file 1)*. STK17b *is a serine/threonine kinase, which has a role in the regulation of apoptosis [23-25]. STK17b is highly expressed in the testes where the apoptosis plays an important role during spermatogenesis. Even though the phenotype of KA-affected boars does not implicate a defect in apoptosis the expression and sequence of *STK17b *mRNA was determined.

### Analysis of the porcine HECW2 gene

The expression pattern of different *HECW2 *fragments (Table 2) appeared to be comparable in the KA-affected and control boar. The full-length mRNA of porcine *HECW2 *[GenBank HM562353] was sequenced from the testis of one KA-affected and one control boar. The total length of the sequenced *HECW2 *transcript was 4802 bp with a high homology with other mammalian species. When compared to the human *HECW2 *gene, the porcine sequence started at position 177 bp in the exon 2. In the pig, exon 1 did not appear to be expressed in the testis. However, based on the genome sequence, exon 1 was highly conserved compared with the human suggesting that it may have an important role in *HECW2 *expression, at least in some tissues. In man, the protein coding region starts at mRNA position 184 bp (exon 2). The human HECW2 protein consists of 1572 aa. Current data suggests that the corresponding pig protein sequence is 1574 aa with a 96% homology to the human sequence. Similarly, the full-length HECW2 protein in the mouse includes 1578 aa and has 95% homology to HECW2 in the pig.

**Table 2:** Primers used for sequencing of the candidate genes HECW2 and STK17b.

Gene	Exons	Position, bp	Length, bp	Forward primer	Reverse primer
*HECW2*	1	-	404	CTGGGACGTGTTTCAAGGTT	GATCTCTGACGCTTGCCTTC
*HECW2*	2-4	1-351	421	AGACGGGATGGCTAGCTCA	GATTTTTATCTCCGGCTCCA
*HECW2*	4-7	332-744	413	AAAAACAGGGGTGTGACTGG	CGGTGCCAGATTGGATTAGT
*HECW2*	5-2-10	483-1534	1052	TGAAGAACCCTGCTGTGATG	GTCTTCCGGCTTTGTCTGAG
*HECW2*	10-12/13	1403-2609	1207	GAGGAAGACCACGAGTTCCA	TACTCTGGTATCGCCGGTTC
*HECW2*	12-15	2543-2896	354	CCGCAGGTGCTGCAGAGGTC	GGTGTCCCGCCGGACTTTGG
*HECW2*	14-20/21	2783-3560	778	TTCCTCATCAGCCCAGAGTT	GCTGAGTACCTGGCGAGTTC
*HECW2*	20-22/23	3461-3800	340	ATGTCATACGTGCCTCCACA	CACTGTAATCCAGCCCTTCC
*HECW2*	21/22-30	3654-4675	1022	AAGGCCCAGGGAAATTAAAG	GGATGGGTATGGAGGGAGAT
*HECW2*	28-	4567-4815	249	AGGGAGTAATGGCCCAAGAA	CTAGAGGGCAGCTTCTGGA
*STK17b*	1-8	1-927	928	GTAAGCTCCGGTCTCCGTCT	TGTTGCTGTGGTAATAGGATCATA
*STK17b*	3-9	413-1100	707	TTTGCTGTGGTTAGGCAATG	AAAAGCCTCTGGATGAAGTCTGT

The exons (based on human AB037722), position of the fragment within the *HECW2 *[GenBank HM562353] and *STK17b *[GenBank HM594868] mRNA and the length of the PCR amplicons are shown.

**Figure 4:**
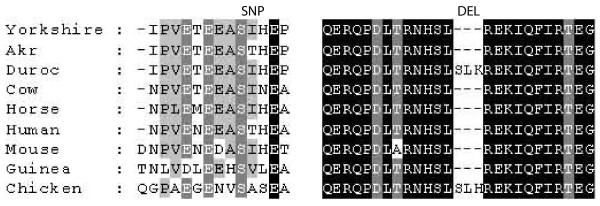
**Alignment of HECW2 protein sequences at the detected polymorphism positions in various species**. The only difference between control (Yorkshire) and KA-affected (Akr) animals was a change from isoleucine to threonine (SNP). However, this change was also present in the human protein sequence. Furthermore, a three aa deletion was identified when compared with the pig reference sequence.

Sequencing of the porcine *HECW2 *mRNA and the exon 1 from genomic DNA of a KA-affected and control boar showed two SNPs at mRNA positions 1563 (SNP1) and 2233 bp (SNP2). SNP1 causes a change in the protein sequence at position 519 aa from isoleucine to threonine (Figure 4). This SNP was further genotyped for all 14 KA-affected and 10 control boars. All KA-affected boars were homozygous for this SNP, but also four control boars had the same homozygous allele, discounting this as the causal mutation of the KAD. In addition to these two SNPs, a deletion of nine bp (three aa) was detected after the nucleotide at position 3348 bp (1113 aa) when compared to the porcine reference sequence [Ensemble: ENSSSCG00000016068]. This deletion seems to be very common in mammalian species (Figure 4). The porcine reference sequence for *HECW2 *included exons 3-28 (based on human [GenBank AB037722]), however our sequencing results indicate that exons 2, 29 and 30 are also expressed in the pig testes.

### Analysis of STK17b mRNA

The sequenced testicular mRNA of porcine *STK17b *[GenBank HM594868] contained exons 1-9 (based on human exon numbering, [GenBank NM_004226]) and 1102 bp. Translation start codon was identified at position 282 within exon 2. No change in the expression profile was identified and the protein coding sequence was identical in the KA-affected and control boar.

## Discussion

While the results of homozygosity mapping of the KAD in a previous study [15] were not statistically significant, they did indicate the most probable positions of the KAD-associated chromosomal segments. In this study we confirmed the association between KAD and porcine chromosome 15. The initial genome screen with microsatellite markers S0004 and SW2608 showed increased homozygosity in KAD affected boars [15]. The genome scan with PorcineSNP60 Genotyping BeadChip (Illumina) localized the KAD associated region between these two markers on porcine chromosome 15. The PorcineSNP60 BeadChip illustrated a high call rate (<95%) in the Finnish Yorkshire pig population and only 15% of the SNPs were monomorphic. In this study we detected the KAD associated region covering 2 Mbp indicating that the marker map in the initial screen was not dense enough to detect the significant increase in homozygosity.

Within the associated region we identified and sequenced two candidate genes *Ubiquitin-protein ligase E3 (HECW2) *and *serine/threonine kinase 17b (STK17b)*. The sequencing of these two genes revealed two SNPs within *HECW2 *gene, but no polymorphisms were detected in the protein coding sequence of *STK17b*. Although the identified mutations appeared not to be the causal cause for the KAD, HECW2 remains a good candidate gene for this defect considering its role in acrosome development and chromatin remodelling.

Protein ubiquitination is one of the fundamental regulatory post-translational modifications controlling intracellular signalling events. Ubiquitin-proteosome-dependent proteolysis plays an important role in selectively degrading and recycling proteins in many basic cellular processes including spermatogenesis [26]. For degradation by the proteosome, binding of ubiquitin with substrate proteins requires the activity of ubiquitin-activating enzyme E1, ubiquitin-conjugating enzyme E2, and substrate-specific ubiquitin ligase E3 [27]. Ubiquitin ligase E3 in combination with an E2 ubiquitin-conjugating enzyme causes the attachment of ubiquitin to a lysine residue on the target protein.

In spermatogenesis ubiquitination is required for various processes; for example the replacement of the spermatids nuclear histones with protamines during spermatid elongation [26]. In spermatozoa, proteosomes are located on the plasma membrane overlying the acrosome, in the acrosomal and postacrosomal regions, in the head-tail connecting-piece, middle-piece of the tail, and residual bodies [28-32]. Proteosome subunit Psmc3 and an ubiquitin protein ligase Rnf19a have been located at the cytosolic side of outer and inner membranes of the acrosome [33]. The co-immunoprecipitation and localization of Psmc3 and Rnf19a in spermiogenesis points to the participation of the ubiquitin-proteosome system in acrosome formation, spermatid head shaping, and development of the head-tail coupling apparatus and tail [33].

Malfunction of components in ubiquitination system has been shown to be a cause of male infertility [27,34-36]. There appears to be a special requirement for certain components of the ubiquitin system during spermiogenesis, in particular [37], and it is probable that different spermatogenic phases would require different specialized activities of the ubiquitin system. Mutations in ubiquitination related proteins may also affect specifically spermatogenesis through their testis specific interacting partners [36]. A malfunction of ubiquitination may cause diverse phenotypes as exemplified in the human and mouse by mutation of *Hr6b *and *Usp14 *[35,38].

## Conclusions

In this study we demonstrate the exact KAD phenotype in mature sperm. In addition to the acrosome, the spermatid chromatin is also affected. We have located the homozygous region for KAD within 0.5 Mbp on porcine chromosome 15 containing two genes *STK17b *and *HECW2*. The role of ubiquitination in chromatin remodelling and acrosome formation is consistent with *HECW2 *being involved in this defect. While a causal mutation for KAD was unable to be identified, our results indicate that the observed phenotype may be caused by a malfunction in the ubiquitination system. Identification of the causal variation for the KAD requires further analysis of the genomic region containing the *HECW2 *gene.

## Methods

### Animal material

Experimental material included 14 Finnish Yorkshire boars affected with KAD and 21 control boars. All affected boars were clinically examined and shown to display symptoms typical of the syndrome, but no other abnormalities. Sperm from affected and control boars was collected and the DNA obtained following phenol/chloroform extraction. Samples were diluted to 100 ng/μl in TE-buffer and used as templates for PorcineSNP60 Genotyping BeadChip (Illumina). Genomic DNA was also used for sequencing of the *HECW2 *exon 1 and SNP1.

For microscopical examination representative semen samples from KA-affected and control boars were fixed in formaldehyde for confocal laser scanning and transmission electron microscopy analyses.

### Confocal laser scanning microscopy

Spermatozoa were labeled with LIVE/DEAD Reduced Biohazard Viability kit (red, L23102, Invitrogen). The labelling protocol was in accordance with the recommendations of the manufacturer. In brief, 50 μl DMSO was added to one vial of fluorescent dye and thoroughly mixed to make a stock solution. Spermatozoa were suspended in PBS at approximately 1 × 10^6^/ml. One μl of fluorescent dye was added to the suspension. After 30 min incubation at room temperature spermatozoa were washed and resuspended in 1 ml PBS twice. One drop of suspension was put on a Superfrost slide and coverslipped and subsequently analyzed on a BioRad MRC 1024 confocal laser scanning microscope. Three-dimensional reconstructions were preformed using Volocity LE free software (http://www.improvision.com).

### Transmission electron microscopy (TEM)

Cells were fixed in 2.5% glutaraldehyde in PBS (pH 7.2) for 2 hours at 4°C. After washing, samples were postfixed in 1% OsO_4 _and 0.5% K-ferrocyanide in PBS for 2 hours, dehydrated with a graded series of acetone, and embedded in Spurr's mixture. Semithin sections were stained by 0.5% toluidine blue (pH 8.5) Areas of interest were trimmed out by comparing the cut surface of the blocks with the semithin sections. Ultrathin sections were cut by an RMC MT-7 ultramicrotome, stained with 2% uranylacetate and lead citrate and analyzed on Philips CM10 electron microscope.

### Genotyping

For high throughput genotyping DNA samples were analyzed by PorcineSNP60 Genotyping BeadChip (Illumina Ltd, San Diego, USA) in the Institute for Molecular Medicine Finland (FIMM, Helsinki, Finland). The PorcineSNP60 BeadChip has recently been developed as an outcome of the porcine whole genome sequencing project [39]).

### Expression profiling and sequencing of STK17b and HECW2

For sequencing the full-length mRNA of the candidate genes *STK17b *and *HECW2*, samples of testicular tissue from a KA-affected and a control boar were collected and stored in RNAlater buffer (Qiagen). Total RNA purification was performed with RNeasy Protect Mini kit (Qiagen). Extracted RNA was reverse transcribed (RT-PCR) using oligo T primers and an ImProm-II Reverse Transcription System (Promega) according to the manufacturer's instructions and amplified using gene specific primers (Table 2). Expression of gene fragments was assessed by gel electrophoresis. For sequencing the PCR amplicons were purified using ExoSAP-IT™ (Amersham Biosciences), while PCR fragments were sequenced in both directions with the same primers used in the amplification procedures. Sequencing was performed on MegaBace 500 capillary DNA sequencer (Amersham Biosciences) using DYEnamic ET Terminator Kits with Thermo Sequenase™ II DNA Polymerase (Amersham Biosciences).

### Statistical analysis

A recessive mode of inheritance was tested for each SNP separately. The recessive model was selected because the pedigree of KA-affected boars suggested a recessive mode of inheritance and the low frequency of the defect in the Finnish Yorkshire pig population. In the recessive model, for each SNP the frequency of homozygote animals for the minor allele (or for the major allele) was compared to frequency of heterozygote and other homozygote animals between cases and controls. In order to correct for multiple testing a permutation procedure was adapted to create empirical genome-wide P-values. Association tests and permutation were carried out using the software package Plink [40]. Haplotypes, the linkage disequilibrium plot and the Manhattan plot were produced with Haploview [41].

## Authors' contributions

AS carried out the molecular genetics studies, sequence alignments and drafted the manuscript. PU performed the statistical analysis and participated in drafting the manuscript. SN participated in the microscopical studies and contributed to drafting of the manuscript (microscopical studies). SP carried out the microscopical studies. MA participated in the design and coordination of the study. JV participated in the design and helped to draft the manuscript. All authors read and approved the final manuscript.

## Supplementary Material

Additional file 1 **The KAD associated region in the Finnish Yorkshire pig localized on porcine chromosome 15**. The KAD associated homozygous region in the Finnish Yorkshire was identified in chromosome 15 between base pairs 93723216 and 94155055. A promising candidate gene *Ubiquitin-protein ligase E3 *(*HECW2*) is located within this region at the same position as two markers with lowest P-values (highlighted by circles). Another gene *STK17b *was also located within the homozygous region; however the known function of this gene in apoptosis would not infer a role for *STK17b *in KAD. Click here for file
