# Supplementary figures and images for: Knobbed acrosome defect is associated with a region containing the genes STK17b and HECW2 on porcine chromosome 15
Source: BMC Genomics. 2010 Dec 9;11:699. doi: 10.1186/1471-2164-11-699 (PMC3016419; doi:10.1186/1471-2164-11-699)

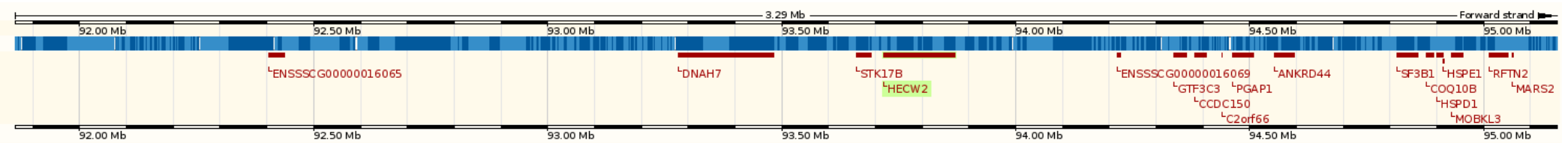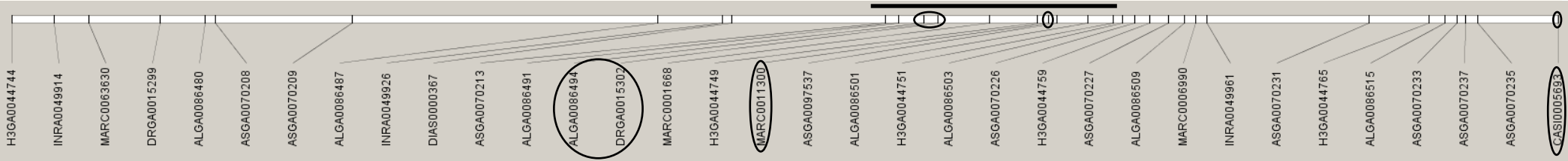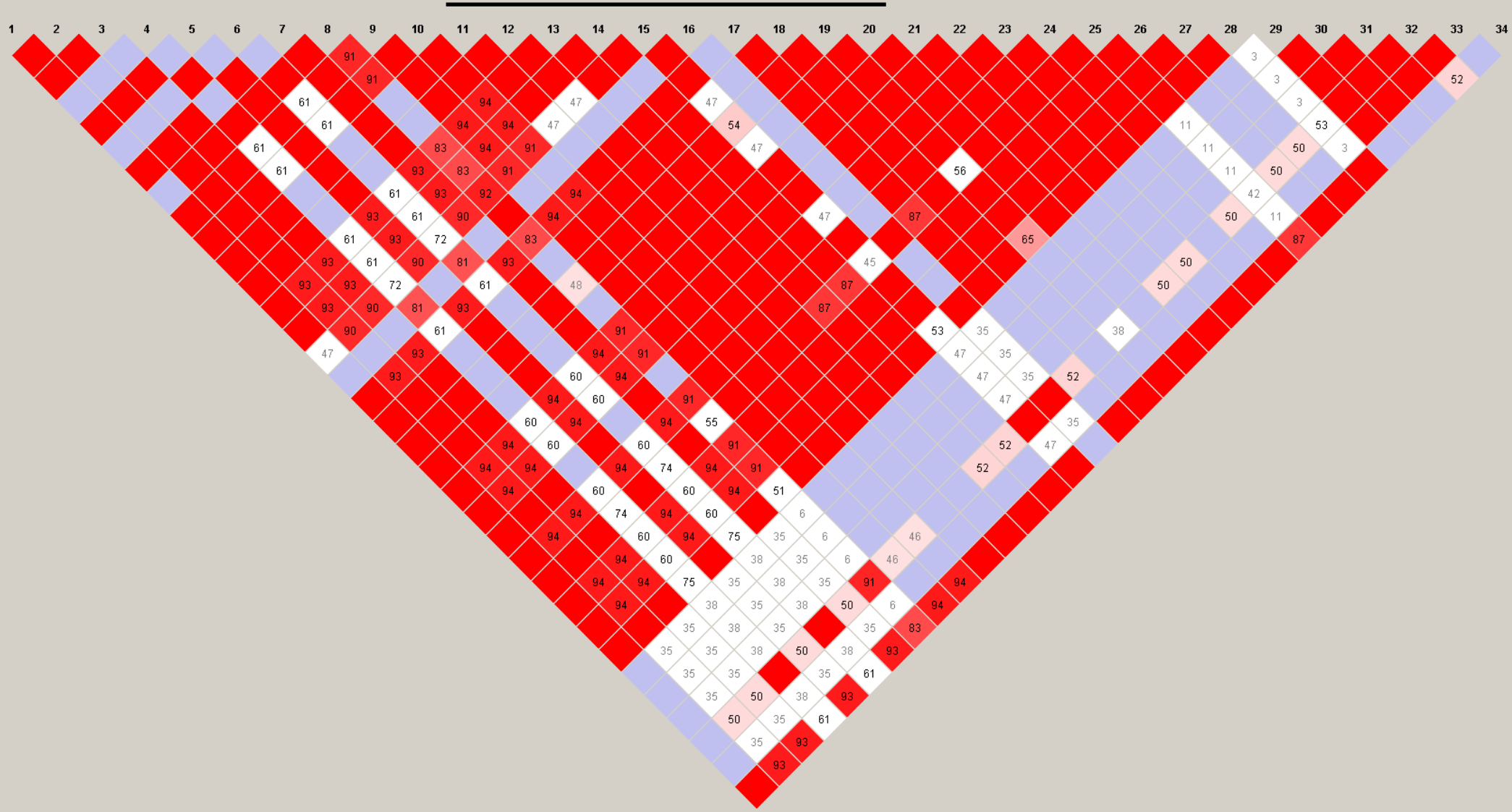

Supplement: Additional file 1 — The KAD associated region in the Finnish Yorkshire pig localized on porcine chromosome 15. The KAD associated homozygous region in the Finnish Yorkshire was identified in chromosome 15 between base pairs 93723216 and 94155055. A promising candidate gene Ubiquitin-protein ligase E3 (HECW2) is located within this region at the same position as two markers with lowest P-values (highlighted by circles). Another gene STK17b was also located within the homozygous region; however the known function of this gene in apoptosis would not infer a role for STK17b in KAD. [file 1471-2164-11-699-S1.PDF]
